# Narrowing band gap and enhanced visible-light absorption of metal-doped non-toxic CsSnCl_3_ metal halides for potential optoelectronic applications†

**DOI:** 10.1039/c9ra10407k

**Published:** 2020-02-24

**Authors:** Jakiul Islam, A. K. M. Akther Hossain

**Affiliations:** Department of Physics, Bangladesh University of Engineering and Technology Dhaka-1000 Bangladesh jakiul.pust.phy.39@gmail.com akmhossain@phy.buet.ac.bd

## Abstract

Non-toxic (lead-free) inorganic perovskites have seized the leading position in the race for the commercialization of solar cells and other photovoltaic devices. The present study is the first theoretical approach to show that metal (Cr/Mn)-doped CsSnCl_3_ perovskites exhibit high optical absorption, high photoconductivity, and high dielectric constant not only in the visible but also in the ultraviolet region of light energy due to the narrowing band gap. We carried out density functional theory (DFT) investigations to find the structural, electronic, optical, and mechanical properties of pristine CsSnCl_3_, Cr-, and Mn-doped CsSnCl_3_ samples in detail. The investigation of the optical functions displayed that the absorption edges of both Cr- and Mn-doped CsSnCl_3_ shifted greatly in the direction of the low photon energy area (red-shift) compared with the pristine sample. An extra very high intensity peak of absorption was noted for both Cr- and Mn-doped CsSnCl_3_ in the visible energy region. The investigation of the mechanical parameters revealed that both Cr- and Mn-doped CsSnCl_3_ samples were as mechanically stable and highly ductile as the pure CsSnCl_3_ sample. The investigation of the electronic properties demonstrated that the creation of intermediate states in the band gap for both the Cr- and Mn-doped CsSnCl_3_ samples made the transition of excited photoelectrons to the conduction band from the valence band easier. A combined study suggested that Mn-doped CsSnCl_3_ was better suited for applications in high potency solar cells and other optoelectronic devices than the other inorganic metal halide perovskites.

## Abstract 2

Non-toxic (lead-free) inorganic perovskites have seized the leading position in the race for the commercialization of solar cells and other photovoltaic devices.

## Graphical abstract

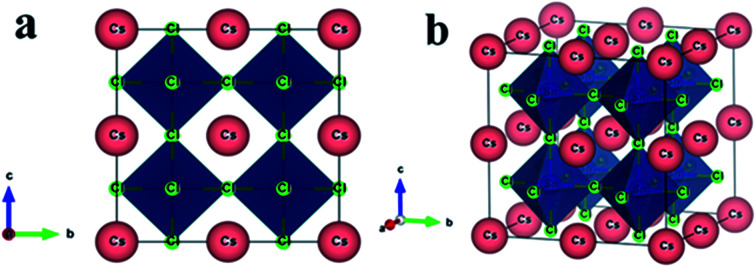

## 1. Introduction

Perovskite-type semiconducting materials with the formula ABX_3_ (where, A = a positive ion, B = a metal ion, and X = a halogen anion) have attracted a huge amount of attention from researchers due to their prominent solar cell efficiency and outstanding optoelectronic features, such as high optical absorption, extended charge diffusion, tunable band gap, tiny carrier effective mass, and high charge bearer mobility.^1,2^ The applications of these metal halide semiconductors are also broad in the area of electronic devices, like photodetectors, light-emitting diodes (LEDs), and solar-to-fuel energy transformation.^3–6^ In addition, these perovskites are inexpensive and abundantly available on earth. Therefore, these semiconducting metal halides are more efficient and economical than the current silicon-based photovoltaic (PV) technology used in solar cell applications.^1^ However, the majority of these metal halide perovskites that reveal excellent features contain lead (Pb). A major concern thus arises for the practical applications of these halide materials in devices due to the toxicity of Pb. Under environmental circumstances, these Pb-based perovskite materials decompose to PbI_2_, which is noxious for the environment.^7–9^ Therefore, several experimental and theoretical investigations have been executed in recent years by replacing Pb with an appropriate metal cation.^10,11^ The investigation of the mechanical properties reported in one theoretical simulation^10^ revealed that the Pb-free perovskite CsSnCl_3_ is ductile in nature and hence suitable for real practical purposes, but the perovskite showed a wide band gap value of 2.8 eV.^12^ The CsSnCl_3_ perovskite is not preferable for solar cell applications due to this broad band gap value. The inclusion of a suitable dopant in CsSnCl_3_ can decrease the band gap value and hence can considerably enhance the optical absorbance in the visible-light-energy region. Therefore, in the present investigation, our aim was to dope various transition metals at the Sn-site of CsSnCl_3_ to enhance the optical absorbance across the whole area of the solar spectrum. Recently, Wu *et al.* experimentally studied the structural and electronic influences of metal substitution at the Sn-site of the CsSnCl_3_ metal halide for the enhancement of light emission.^13^ Zhang *et al.* recently investigated the defect properties of pure CsSnCl_3_ perovskite and they reported very crucial information about the fabrication and also further modulation of the perovskite.^14^ In 2018, metal-doped CsGeCl_3_ was reported in a theoretical work,^15^ but the metal-doped CsGeCl_3_ gave a Poisson's ratio value of 0.26, which is the same as the critical value (0.26) of Poisson's ratio needed to ensure a separation between the ductile and brittle behavior of a material and, as a result, metal-doped CsGeCl_3_ showed lower ductility than pristine CsGeCl_3_. Therefore, metal-doped CsGeCl_3_ may not be suitable for some practical applications where high ductility is demanded. Another metal halide CsGeI_3_ perovskite showed a high optical absorption, as investigated by Roknuzzaman *et al.*, but CsGeI_3_ is brittle in nature and hence not appropriate for real practical purposes.^10^ Therefore, in the present work, we studied the structural, electronic, optical, and mechanical properties of Cr- and Mn-doped CsSnCl_3_ samples to find a better lead-free highly ductile metal halide material with enhanced optical absorption, ameliorated photoconductivity, and a high dielectric constant for an optimum potency solar cell and other optoelectronic applications than the previously reported well-entitled perovskite CsGeI_3_ and metal-doped CsGeCl_3_ samples. Finally, a detailed comparative analysis was performed among the different key properties of pure and metal-doped CsSnCl_3_ with the metal-doped CsGeCl_3_ and the best known CsGeI_3_ metal halide.

## 2. Computational details

*Ab initio* density functional theory^16,17^ simulations were carried out with the help of a plane wave pseudopotential approach in the CASTEP (Cambridge Serial Total Energy Package) code of Materials Studio-7.0.^18,19^ The ultrasoft pseudopotential^20^ of a Vanderbilt type was used for the description of the electron ion interactions. GGA (Generalized Gradient Approximation) was employed in the advance form of PBE (Perdew–Burke–Ernzerhof)^21^ to evaluate the exchange–correlation energy. Pseudo-atomic calculations were executed only taking the valence electrons into consideration. The influence of metal-doping on the cubic CsSnCl_3_ perovskite was studied by constructing a 2 × 2 × 2 supercell containing 40 atoms. As a consequence, the metal-doped CsSnCl_3_ material possessed the new chemical formula CsSn_1−*x*_M_*x*_Cl_3_ (*x* = 0.125 and M = Cr, Mn). A plane wave energy cutoff of 550 eV and 14 × 14 × 14 *k*-points for a pure single cell and an energy cutoff of 350 eV and 3 × 3 × 3 *k*-points for the pure supercell and doped metal halide perovskites were used for both the geometry optimization and electronic property investigation. The Monkhorst–Pack technique^22^ was used for sampling the *k*-points over the first Brillouin zone. To ensure the optimized configuration in the crystal structure, the BFGS (Broyden–Fletcher–Goldfarb–Shanno) technique^23^ was employed. Elastic constants for both the pure and doped samples were computed with the help of finite strain theory^24^ in the CASTEP module, setting 0.003 as the optimum value of strain amplitude. The mechanical properties were found out using the VRH (Voigt–Reuss–Hill) averaging method^25^ from the computed value of *C*_*ij*_. A scissor value of 1.832 eV was used during the optical property computations in order to reduce the band gap between the theoretical value (0.968 eV) and experimental value (2.8 eV) of the pure CsSnCl_3_ band gap. The criteria of convergence thresholds for the geometry optimization were set as follows: (i) total energy, 5 × 10^−6^ eV per atom, (ii) maximum stress, 0.02 GPa, (iii) maximum force, 0.01 eV Å^−1^, and (iv) maximum displacements, 5 × 10^−4^ Å.

## 3. Results and discussion

### 3.1. Structural properties

**Fig. 1:**
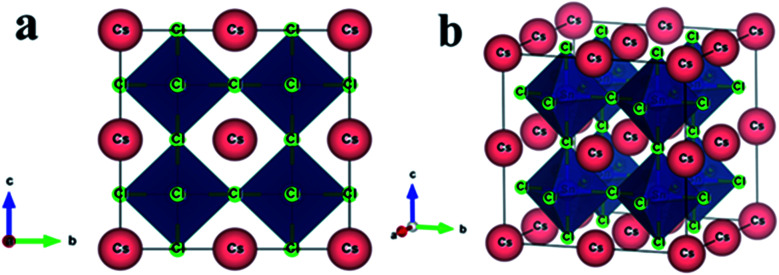
The constructed crystal structure (2 × 2 × 2 supercell) of the CsSnCl_3_ metal halide. (a) Two-dimensional and (b) three-dimensional views.

Non-toxic semiconducting metal halide CsSnCl_3_ perovskite possesses a cubic crystal structure with the space group *Pm*3̄*m* (no. 221).^10^ The unit cell of the halide perovskite contains five atoms in a single formula unit. The Wyckoff positions 1a, 1b, and 3c with fractional coordinates (0, 0, 0), (0.5, 0.5, 0.5), and (0, 0.5, 0.5) are occupied by Cs, Sn, and Cl atoms, respectively.^10^ The constructed supercell of CsSnCl_3_ to dope a transition metal is eight times larger than the pristine sample in size for containing atoms and the supercell consists of 40 atoms comprising 8 Cs atoms, 8 Sn atoms, and 24 Cl atoms, as shown in Fig. 1.

The impurity added in the pure supercell of CsSnCl_3_ by replacing one Sn atom by Cr/Mn gave a concentration of doping of about 12.5%. The computed values of the lattice parameter and unit cell volume of the pristine and doped perovskites in this work are compared with the available experimental and other theoretical works tabulated in Table 1. The calculated values of the unit cell volume and lattice parameter of the pristine sample in this work were highly consistent with the values observed in earlier experimental works^11,26^ and approximately near to the other theoretical works.^10,27^ This indicates the reliability of our DFT-based study. The computed values of the unit cell volume and lattice parameter of the doped perovskite were reduced due to the lower value of the ionic radii of the dopant elements Cr^2+^ (0.73 Å) and Mn^2+^ (0.67 Å) compared to Sn^2+^ (1.10 Å).^28,29^

**Table 1:** The computed and the available theoretical and experimental lattice parameters and the calculated unit cell volumes of pristine and metal-doped CsSnCl_3_ samples

Phase	*a* (Å)			*V* (Å^3^)
	This work	Other works	Experimental	
CsSnCl_3_	5.620	5.613^a^, 5.610^b^	5.560^c^, 5.573^d^	177.504
CsSn_1−*x*_Cr_*x*_Cl_3_	5.546	—	—	170.585
CsSn_1−*x*_Mn_*x*_Cl_3_	5.540	—	—	170.031

a From ref. 10.

b From ref. 27.

c From ref. 26.

d From ref. 11.

### 3.2. Optical properties

The optical functions of a substance provide crucial information about the interaction nature of the substance with light energy. The optical functions are highly crucial criteria to gain deep knowledge about the electronic configuration of the material and subsequently the practical uses of the material in photovoltaic devices, such as solar cells. The non-toxic CsSnCl_3_ perovskite possesses medium optical absorption and less optical conductivity and hence pure CsSnCl_3_ is not preferable for application in solar cells.^10^ Therefore, we studied in detail the important optical functions, including the optical absorbance, conductivity, real and imaginary parts of the dielectric functions, and the reflectivity of the pristine and metal-doped (Cr and Mn) CsSnCl_3_ to find a better candidate for solar cells and other potential optoelectronic applications. In the theoretical approach to find various optical functional materials, 0.5 eV Gaussian smearing is typically used because at that smearing, the *k*-points on the Fermi surface become sufficiently effective.

**Fig. 2:**
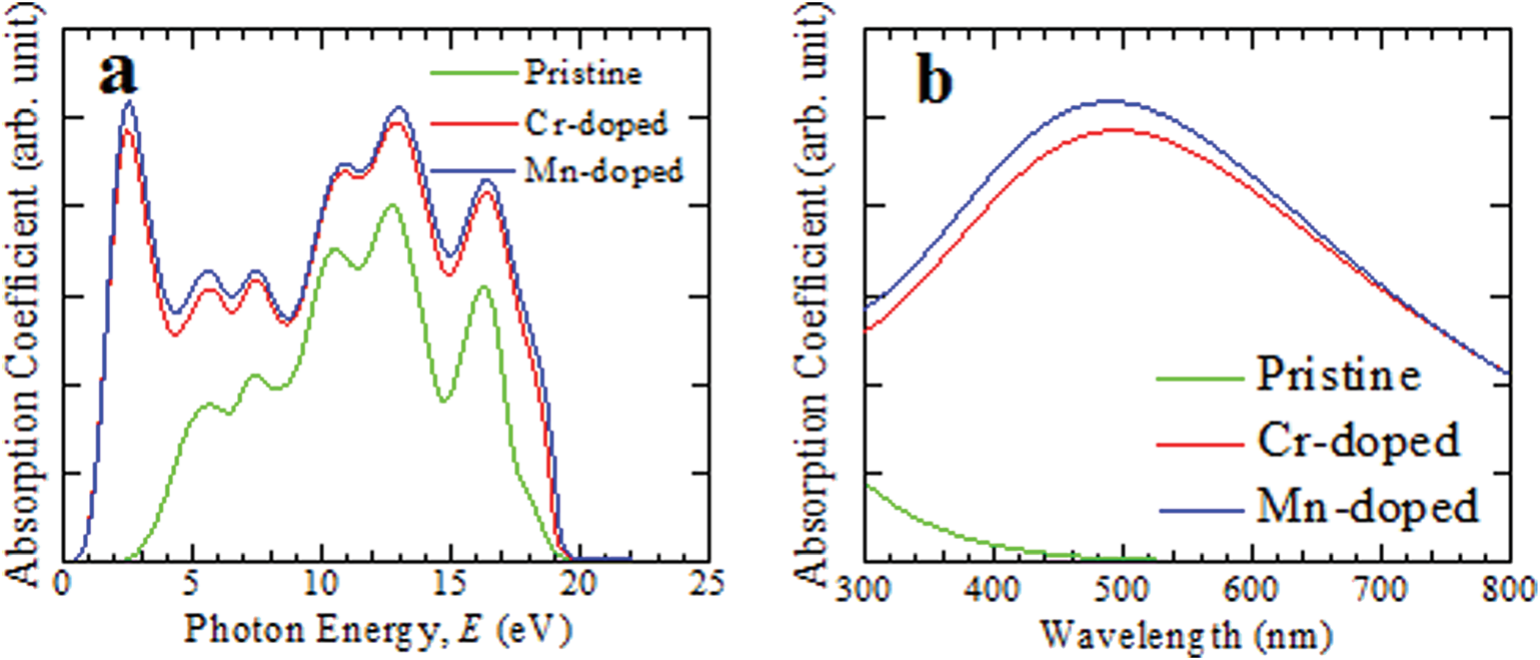
Simulated light absorption spectra of intrinsic and transition metal-doped CsSnCl_3_ materials, (a) as a function of light energy and (b) as a function of the wavelength of light.

The investigated optical absorbance spectra of the pristine and doped CsSnCl_3_ perovskites are presented in Fig. 2. When light at a particular wavelength (energy) penetrates a material then its intensity goes on decreasing and the penetration of light before its absorbed is assessed by the absorption coefficient. It is a highly crucial parameter to know about the ability of a material to absorb incident light and this provides a crucial idea about the capability of a material for achieving optimum solar energy conversion, which is useful for the application of a material in remarkable performance photovoltaic devices, such as solar cells. The photon energy-dependent absorption spectra of both the intrinsic and doped CsSnCl_3_ are illustrated in Fig. 2(a). From Fig. 2(a), it is explicit that the absorption terminal of both the Cr- and Mn-doped CsSnCl_3_ had been shifted greatly in the direction of the low photon energy zone (red-shift) compared with the pristine sample. The Cr- and Mn-doped CsSnCl_3_ exhibited a very high absorption co-efficient in all the energy levels between 0.34 eV and 20 eV, as illustrated in Fig. 2(a). An additional very high peak of absorption was noted at ∼2.57 eV (visible region) for both Cr- and Mn-doped CsSnCl_3_. The Mn-doped CsSnCl_3_ exhibited higher absorption peaks than the Cr-doped sample and the reason behind the high absorptivity of the Mn-doped sample than the Cr-doped CsSnCl_3_ is explained in the electronic properties portion.

The pristine CsSnCl_3_ revealed no absorption peak in the visible region. The absorption co-efficient was enhanced in a very high way not only in the low energy area (visible region) but also in the high energy area (ultraviolet region) due to the metal-doping in the pure CsSnCl_3_ sample. The absorption coefficient as a function of wavelength of light is illustrated in Fig. 2(b) for further understanding the light absorbance phenomenon of intrinsic and metal-doped CsSnCl_3_ in the visible zone. The Mn-doped CsSnCl_3_ perovskite revealed a wider absorption area than the Cr-doped CsSnCl_3_, whereas pure CsSnCl_3_ revealed approximately zero absorbance in the visible region. Generally, semiconductors with a wide band gap are capable of absorbing ultraviolet (UV) light of the solar spectrum, which only represents about 4% of the whole solar energy arriving at the earth.^30^ The visible light includes nearly about 43% of the solar spectrum.^31^ Therefore, the pure CsSnCl_3_ (band gap = 2.80 eV) is not capable of utilizing the visible-light-energy portion of the solar spectrum and hence pure CsSnCl_3_ is not suitable for use in solar cells. The Mn-doped CsSnCl_3_ showed a more prominent absorption of visible light as well as UV light of the solar spectrum than the Cr-doped sample. Therefore, the Mn-doped CsSnCl_3_ can be the best candidate for the proper utilization of the solar spectrum and may increase the performance of solar cells greatly.

**Fig. 3:**
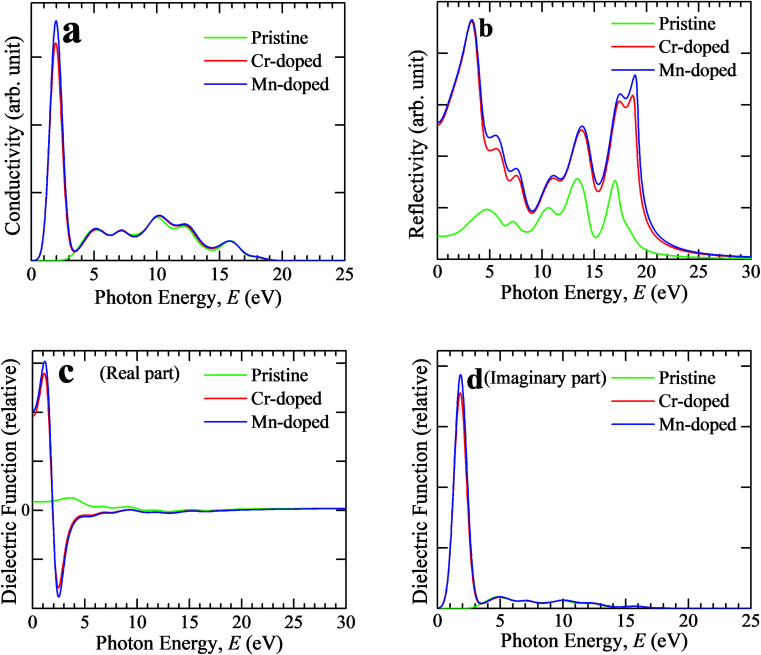
Computed spectra of (a) conductivity, (b) reflectivity, (c) real portion of the dielectric function, and (d) imaginary portion of the dielectric function of pure and metal-doped CsSnCl_3_ samples as a function of incident light energy.

Optical conductivity is also specified as photoconductivity. The photoconductivity and hence electrical conductivity increase due to the increase in absorbing photons. The conductivity spectra (real part) are shown in Fig. 3(a) up to 25 eV photon energy for both the pristine and transition metal-doped CsSnCl_3_ samples. The optical conductivity of the pristine sample was approximately similar to both Cr- and Mn-doped CsSnCl_3_ in the high-energy region (above 3.3 eV), but an additional very sharp peak could be observed in the low-energy region (visible area) for both the metal-doped CsSnCl_3_ samples. Mn-doped CsSnCl_3_ revealed a higher conductivity than Cr-doped CsSnCl_3_. The observed high optical conductivity in the low-energy region for both doped CsSnCl_3_ samples, as illustrated in Fig. 3(a), was a result of their high absorptivity in the visible region (Fig. 2).

Reflectivity is an important optical parameter that can provide crucial information for determining the photovoltaic applications of a material. The reflectivity is one kind of assessment of the ability of a material to reflect light energy from the layers of the material. The reflectivity is specified as the amount of reflection of light energy from the material surface compared to the amount of incident light energy on the material surface. Fig. 3(b) illustrates the reflectivity spectra of intrinsic and metal-doped CsSnCl_3_ for light energy up to 30 eV. The pristine CsSnCl_3_ exhibited low reflectivity over the whole solar energy spectrum, whereas the metal-doped samples revealed high reflectivity. An extra peak could be observed in the low-energy region for both doped CsSnCl_3_ samples. The Mn-doped CsSnCl_3_ sample exhibited a higher reflectivity than the Cr-doped sample over the whole range of the solar spectrum as illustrated in Fig. 3(b). Hence, further investigation should be carried out to decrease the reflectivity of the metal-doped CsSnCl_3_, which may further improve the absorbance as well as the performance of the solar cells.

The behavior of a material in contrast to incident photon energy is termed as the dielectric function. The static value of the dielectric constant is a very useful criterion providing prominent information about the charge-recombination rate and the hence the overall efficiency of optoelectronic devices.^32^ A high value of the dielectric constant of a material implies that the material has a comparatively low charge-carrier recombination rate and as a consequence the entire performance of the optoelectronic devices will be improved. The real and imaginary portions of the dielectric function of the pristine and metal-doped CsSnCl_3_ samples are displayed in Fig. 3(c) and (d). The real and imaginary parts of the metal-doped CsSnCl_3_ samples exhibited a very sharp peak for the dielectric function in the low-energy zone, whereas the pristine sample revealed approximately no value of dielectric function compared to the metal-doped samples. Materials that have a wide value of band gap generally show a lower value dielectric constant.^33^ Therefore, the metal-doped samples revealed higher value dielectric constants than the pristine sample as the band gap was decreased (as shown in the electronic properties portion) after doping the transition metals in the pure CsSnCl_3_ sample. The imaginary portion of the dielectric function is directly related to the electronic band structure of a crystal and explains its absorptive behavior.^34^ The appearance of a very sharp peak of the imaginary portion of the dielectric function of the metal-doped CsSnCl_3_ materials in the visible zone implied that both the doped samples had a high absorbance in the visible area, which was also justified by the absorption spectra, as illustrated in Fig. 2(a). The Mn-doped CsSnCl_3_ revealed a higher value of the dielectric function in the low-energy region than the Cr-doped sample, which suggests that the Mn-doped CsSnCl_3_ can be a promising candidate for optoelectronic applications. Both the real and imaginary portions of the dielectric function of the metal-doped CsSnCl_3_ samples were almost similar to the pristine sample in the ultraviolet zone (high energy region). The imaginary portion of the dielectric function reached zero in the high-energy area (above 17 eV), whereas the real portion went almost to unity for all the samples. This simulated result suggests that both the pristine and doped CsSnCl_3_ samples had a high transparency and hence less absorption in the high-energy region (above 17 eV).

### 3.3. Electronic properties

**Fig. 4:**
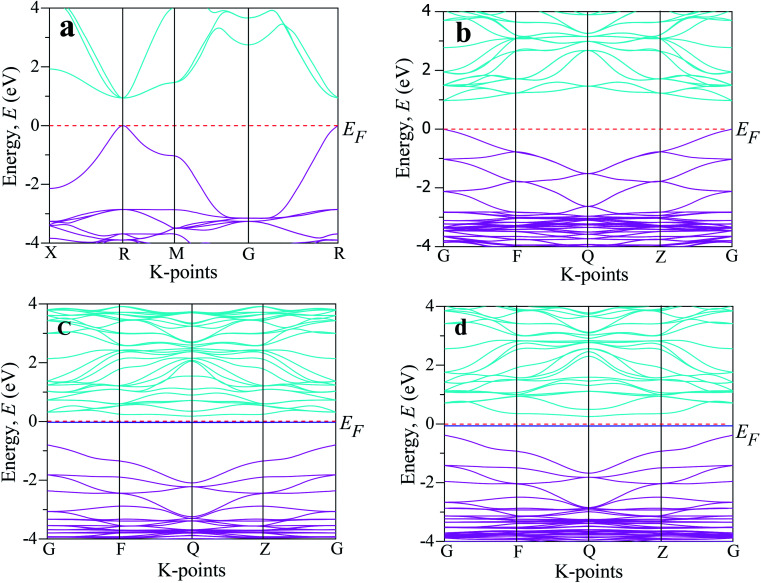
The electronic band structure of CsSnCl_3_ perovskite computed by using: (a) an intrinsic single cell, (b) intrinsic supercell, (c) Cr-doped sample, and (d) Mn-doped sample.

Analysis of the electronic phenomena (such as band structure and DOS) is very fruitful to aid understanding the optical functions properly. The investigated band structures of the intrinsic and transition metal-doped CsSnCl_3_ are demonstrated in Fig. 4. The Fermi level (*E*_F_) for all the samples is presented at zero of the energy scale, which goes from −4 eV to +4 eV. The Fermi level exists below the conduction band and above the valence band. As we know from semi-conductive theory, the physical performance of a solid substance is largely dependent on the energy band structure close to the Fermi level. Therefore, we analyzed the lower portion of the conduction band and the peak of the valence band around the Fermi level. The band structure investigated using a single cell of pure CsSnCl_3_ perovskite is displayed in Fig. 4(a). The lower portion of the conduction band and the peak position of the valence band existed at the R-point and hence the pure CsSnCl_3_ is a direct band gap semiconducting material, as illustrated in Fig. 4(a).

The direct band gap is also exhibited at other symmetry points (X, M, G). The observed band gap using a single cell of pure CsSnCl_3_ at the R symmetry point was 0.943 eV, which is the same as the band gap of pure CsSnCl_3_ reported by Roknuzzaman *et al.*,^10^ highlighting the accuracy of our DFT study. The simulated band structure using the supercell (eight times) of CsSnCl_3_ is presented in Fig. 4(b). The observed band gap between the minimum of the conduction band and the peak of the valence band at the G-point of the Brillouin zone was 0.968 eV (direct band gap), which indicates good consistency with the simulated band gap using the single cell of CsSnCl_3_ perovskite. It was clear that the simulated band gap using GGA underestimates the experimentally observed value of the band gap, 2.8 eV.^12^ This is one of the general limitations of the GGA approach and arises due to the correlation effects and electron–electron exchange approximation. The usual problem of underestimation of the band gap is also observed in LDA (Local Density Approximation) and LDA+U techniques. To overcome this band gap underestimation, researchers have developed some methods, such as the hybrid functional^35^ and GW method,^36^ but these methods also have some limitations. The GGA+U^37^ approach is used to make a partial correction of the band gap of the theoretical value compared to the experimentally assessed value. However, the GGA approach does not affect our comparative analysis of the band structure and the DOS of the pristine and metal-doped CsSnCl_3_ samples. The band structure of Cr-doped CsSnCl_3_ is illustrated in Fig. 4(c) and the simulated band gap between the minimum of the conduction band at the Q symmetry point and the peak of the valence band at the G symmetry point was 0.979 eV (indirect band gap), which was slightly larger than band gap of the pure CsSnCl_3_ sample, albeit an intermediate state was noticed in the band gap. Then, the observed band gap of Cr-doped CsSnCl_3_ between the minimum of the conduction band at the Q symmetry point and the intermediated state was 0.205 eV (indirect band gap), which was so much smaller than that of the pristine CsSnCl_3_.

The creation of intermediate states makes electrons transfer easier under visible light from the valence band to the conduction band. Consequently, the Cr-doped CsSnCl_3_ samples exhibited very high absorbance compared to the pure CsSnCl_3_ sample due to the appearance of the intermediate state. An intermediate state was also noticed in the band gap of Mn-doped CsSnCl_3_ as illustrated in Fig. 4(d), and the assessed indirect band gap was 3.15 eV. In the case of Mn-doped CsSnCl_3_, the conduction band was shifted to lower energy (gets closer to the Fermi level) region relative to the pristine CsSnCl_3_ and the valence band was shifted to the higher energy (gets closer to the Fermi level) region as compared to Cr-doped CsSnCl_3_. The band gap of Mn-doped CsSnCl_3_ between the peak of the valence band (at the G point) and the lower portion (at the Q point) of the conduction band was 0.623 eV (indirect band gap), which was smaller than the simulated band gap of Cr-doped and pristine CsSnCl_3_ and as a consequence, the transfer of electrons to the conduction band from valence band was easier in the Mn-doped CsSnCl_3_ sample than in the cases of the pure and Cr-doped samples. Therefore, the Mn-doped CsSnCl_3_ sample exhibited higher absorptivity in the visible region (Fig. 2) than the pristine and Cr-doped samples and hence Mn-doped CsSnCl_3_ is more suitable for efficient solar cell applications.

**Fig. 5:**
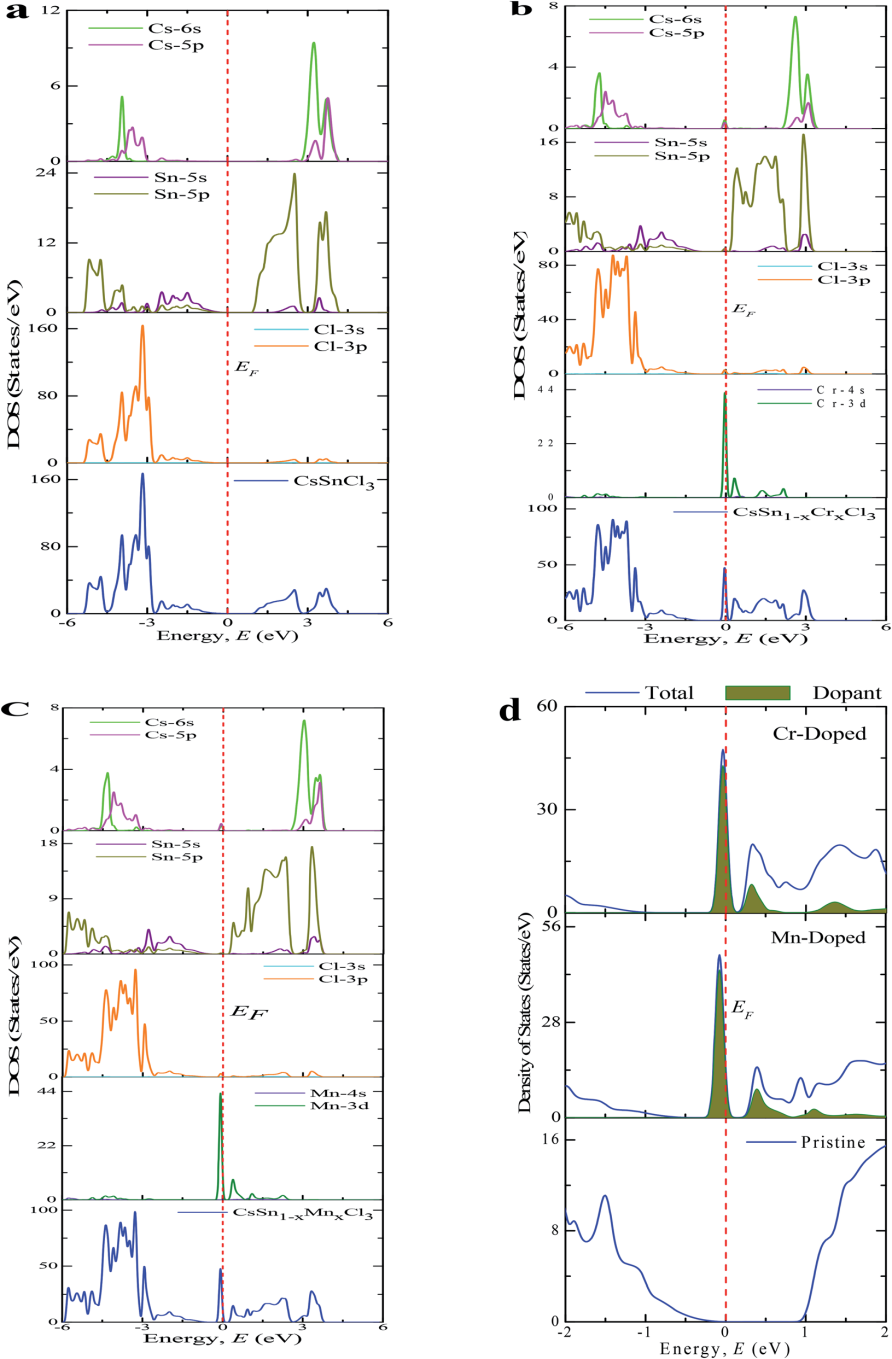
The total and partial density of states of CsSnCl_3_ metal halide computed by using: (a) a pure supercell, (b) Cr-doping at the Sn-site, (c) Mn-doping at the Sn-site, and (d) the dopant contribution close to the Fermi level.

The PDOS (Partial Density of States) and TDOS (Total Density of States) of the pure and doped CsSnCl_3_ samples are displayed in Fig. 5. The valence band (below the Fermi level) of pristine CsSnCl_3_, as exhibited in Fig. 5(a), was mainly composed of a Cl-3p orbital with a little contribution of Sn-5s, Sn-5p, Cs-6s, and Cs-5p orbitals and the conduction band (above the Fermi level) was mostly comprised of a Sn-5p orbital with a little contribution of Cs-6s and Cs-5p orbitals. The density of states (partial and total) of the Cr- and Mn-doped CsSnCl_3_ samples, as illustrated in Fig. 5(b) and (c), respectively, showed that the overall DOS profiles of both the doped compositions were almost similar to the intrinsic sample but the conduction band was shifted slightly toward the lower energy zone for both the doped compositions as well as an extra peak appeared in the valence band near to the Fermi level in both the doped samples due to the creation of dopant states of Cr-3d for CsSn_1−*x*_Cr_*x*_Cl_3_ and Mn-3d for CsSn_1−*x*_Mn_*x*_Cl_3_. To give a clear insight of the dopant states contribution in changing the band gap, the dopant states contribution near the Fermi level are shown in Fig. 5(d). The dopant energy states appeared in the middle of the band gap for both the Cr- and Mn-doped CsSnCl_3_ samples. These intermediate energy states minimize the energy, which is essential for the transfer of excited photoelectrons to the conduction band from valence band. First, the valence electrons are transferred to the intermediate band (dopant energy states) and subsequently transferred to the conduction band under visible-light energy. These results are the reason for the red-shift as well as the high absorptivity of the metal-doped CsSnCl_3_ samples relative to the pristine CsSnCl_3_ as illustrated in Fig. 2.

### 3.4. Mechanical properties

The elastic constants are very crucial toward knowing about a crystal's mechanical stability. Therefore, we computed the elastic constants by the use of finite strain theory^24^ in a CASTEP module. The cubic crystal CsSnCl_3_ consists of three distinct elastic constants (*C*_11_, *C*_12_, and *C*_44_) and the computed values of the elastic constants of the intrinsic and doped phases in this DFT simulation and other theoretical works for pure samples are tabulated in Table 2. A crystal is mechanically stable if it satisfies the well-known stability criteria provided by Born.^38^ The Born stability criteria can be written in the form:*C*_11_ > 0, *C*_44_ > 0, *C*_11_ + 2*C*_12_ > 0 and *C*_11_ − *C*_12_ > 0

**Table 2:** Computed elastic constants *C*_*ij*_ (GPa) and Cauchy pressure (GPa) of the pristine and metal-doped CsSnCl_3_ materials

Phase	*C*_11_	*C*_12_	*C*_44_	*C*_12_–*C*_44_
CsSnCl_3_	49.35	8.77	5.95	2.82
CsSn_1−*x*_Cr_*x*_Cl_3_	51.22	9.73	6.98	2.75
CsSn_1−*x*_Mn_*x*_Cl_3_	52.72	9.81	7.23	2.58
aCsSnCl_3_	50.66	8.71	6.01	—

a From ref. 10.

It is clear from Table 2 that the pristine and doped cubic halide materials were mechanically stable because both the pristine and doped perovskite phases satisfy the above stability criteria. The elastic constants of the intrinsic sample simulated in this DFT investigation are highly consistent with the other theoretical simulation results,^10^ which indicates the accuracy of this DFT simulation.

The Cauchy pressure (*C*_12_–*C*_44_) is a significant criterion that provides an idea about a crystal's brittleness and ductile characteristic.^39^The ductile (brittle) nature of a crystal is represented by a positive (negative) value of Cauchy pressure. In this work, from Table 2, the Cauchy pressure of both the pure and doped samples were positive, which indicates the ductile nature of both the pristine and doped materials. The most prominent mechanical parameters, such as the bulk modulus (*B*), shear modulus (*G*), *B*/*G* ratio, Young's modulus (*E*), and Poisson's ratio (*v*), of the pristine and doped materials were computed using the evaluated elastic constants with the help of the Voigt–Reuss–Hill (VRH) averaging method^25^ and the results are displayed in Table 3. The Voigt and Reuss bounds of the bulk and shear modulus for the cubic crystal were computed using the following relations:1
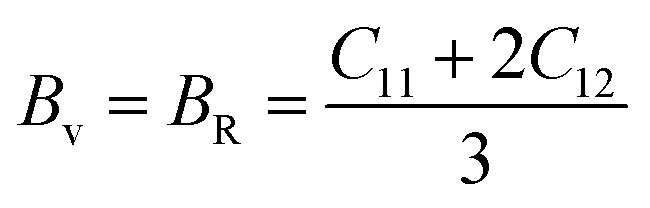
2
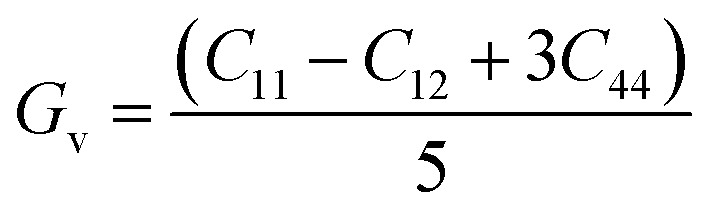
3
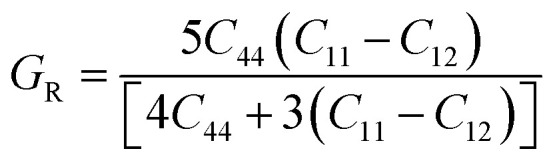


**Table 3:** Calculated bulk modulus (*B*), shear modulus (*G*), Young's modulus (*E*), Pugh's ratio (*B*/*G*), and Poisson's ratio (*ν*) of the pristine and metal-doped CsSnCl_3_ materials

Phase	*B* (GPa)	*G* (GPa)	*E* (GPa)	(*B*/*G*)	*v*
CsSnCl_3_	22.30	10.00	26.10	2.23	0.305
CsSn_1−*x*_Cr_*x*_Cl_3_	23.56	11.00	28.55	2.14	0.298
CsSn_1−*x*_Mn_*x*_Cl_3_	24.11	11.37	29.48	2.12	0.296
aCsSnCl_3_	22.70	10.20	26.61	2.22	0.300

a From ref. 10.

The arithmetic average value of the bulk (*B*) and shear modulus (*G*) were computed with the help of Hill's approximation by using the values of *B*_v_, *B*_R_, *G*_v_, and *G*_R_ as follows:4
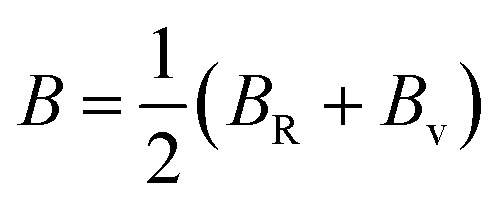
5
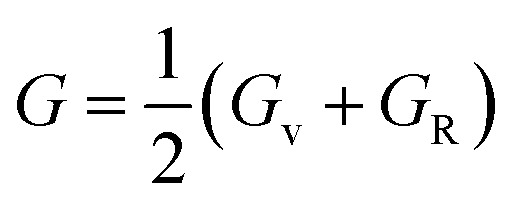


The bulk modulus (*B*) and shear modulus (*G*) were used to find the Young's modulus (*E*) and Poisson's (*v*) ratio by using the following formulas:6
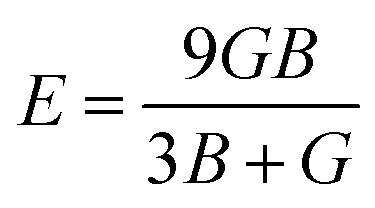
7
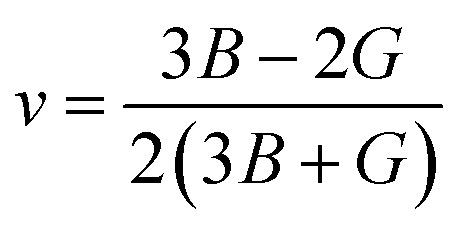


It is clear from Table 3 that all the mechanical parameters of the pristine sample calculated in this work revealed a good consistency with other available theoretical simulations.^10^ The bulk modulus (*B*) is one of the most prominent phenomena that indicate the stiffness of a material. The calculated values of the bulk modulus for both the pristine and doped samples were low, which implied the flexibility and softness of all the samples. The value of the bulk modulus increased slightly after metal doping but it was still too low compared to other metal-doped samples reported in other theoretical work.^15^ The lower values of bulk modulus for both the Cr- and Mn-doped CsSnCl_3_ materials indicated the softness and flexibility of the doped materials.

**Fig. 6:**
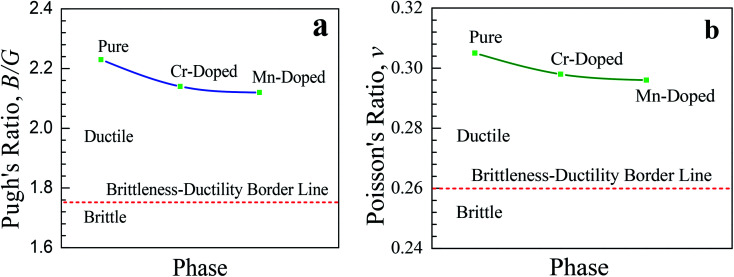
Variation of (a) Pugh's ratio and (b) Poisson's ratio of the pure and metal-doped CsSnCl_3_ metal halide. The broken red line indicates the border between the brittle and ductile behavior of a material.

Therefore, both the doped samples are suitable to make thin films and hence more preferable for various potential optoelectronic applications. A similar affinity was observed for the shear modulus (*G*) and Young's modulus (*E*) of the pristine and doped perovskites. The *B*/*G* ratio, which is commonly known as Pugh's ratio, is a useful parameter that indicates the failure mode, *i.e.*, brittleness and ductility, of a crystal. The critical value of Pugh's ratio to separate between the brittleness and ductility of a material is 1.75.^40^ If the value of the *B*/*G* ratio is lower (greater) than 1.75, then the crystal is considered as brittle (ductile). The studied pristine and doped samples were highly ductile in nature as they had Pugh's ratios greater than the critical value (1.75). Though the value of *B*/*G* decreased slightly after Cr- and Mn-doping in the CsSnCl_3_ perovskites, the value was still far larger than the critical value (1.75) and hence both the doped perovskites were as highly ductile as the pristine one. The Poisson's ratio (*ν*) is another prominent criterion that provides crucial information about the stability and bonding force of a crystal. A value of Poisson's ratio greater than 0.25 and lower than 0.5 indicates the existence of central force in the crystal.^41^ The values of Poisson ratios of the studied pristine and doped perovskite CsSnCl_3_ materials were greater than 0.25, which revealed the presence of central force in all the materials.^42^ The critical value of Poisson's ratio for the separation between the ductile and brittle behavior of a material is 0.26.^10^ If the value of Poisson's ratio is greater (lower) than the critical value, then the crystal is considered as ductile (brittle). The calculated value of Poisson's ratios for all the compositions was ∼0.30, which revealed the highly ductile behavior of both the pristine and doped CsSnCl_3_ perovskites. The variations in the Pugh's ratio (*v*) and Poisson's ratio (*B*/*G*) of the pure and metal-doped CsSnCl_3_ perovskites are illustrated in Fig. 6 for further better understanding the ductile characteristic of the samples. The higher values of *v* and *B*/*G* indicate that both the metal-doped phases were as highly ductile as the pure phase and hence the metal-doped phases are suitable for potential device applications.

### 3.5. Non-toxic perovskites

The inorganic perovskite CsGeI_3_ has shown high absorptivity and photoconductivity and as a consequence is suitable for solar cell and other photovoltaic applications as reported in theoretical work,^10^ but the CsGeI_3_ metal halide reveals a brittle behavior and hence is not appropriate for practical application in devices. Recently, metal-doped CsGeCl_3_ was investigated by DFT simulation^13^ in order to find a material of high absorptivity and conductivity for high-efficiency solar cells and other optoelectronic applications, but the metal-doped CsGeCl_3_ had a Poisson's ratio value of 0.26, which is the same as the critical value (0.26) of Poisson's ratio for the separation between the ductile and brittle behavior of a material and as a consequence the metal-doped CsGeCl_3_ showed less ductility than the pristine CsGeCl_3_. Therefore, the metal-doped CsGeCl_3_ may not be suitable for some practical applications where high ductility is demanded. Investigation of the mechanical properties reported by Roknuzzaman *et al.*^10^ showed that the lead-free perovskite CsSnCl_3_ was ductile in nature and hence suitable for real practical uses, but the perovskite revealed a wide band gap value of 2.8 eV (ref. 12) and as a result, CsSnCl_3_ showed only medium optical absorption and photoconductivity. Therefore, the pure CsSnCl_3_ perovskite is not preferable for solar cell applications. A suitable metal dopant in CsSnCl_3_ can solve this problem however. According to our theoretical work, our investigation of the mechanical parameters revealed that both the doped samples were highly ductile and exhibited lower values of bulk modulus as the pristine CsSnCl_3_ and hence these samples are suitable to make thin films and as a consequence, more efficient in various optoelectronic applications, such as solar cells. Metal (Cr/Mn) doping at the Sn-site of CsSnCl_3_ enhanced the absorptivity as well as the photoconductivity to a greater extent not only in the visible but also in the ultraviolet region of light energy. However, Mn-doped CsSnCl_3_ exhibited a higher absorptivity as well as photoconductivity than the Cr-doped sample. From the above study, we can say that Mn-doped CsSnCl_3_ is the best non-toxic (lead-free) metal halide perovskite for solar cells and other potential photovoltaic applications among the inorganic metal halide perovskite materials.

## 4. Conclusions

In brief, the structural, electronic, optical, and mechanical properties of pure and metal-doped CsSnCl_3_ were investigated using the DFT-based first-principles calculations. Our investigation of the mechanical properties demonstrated that both the Cr- and Mn-doped CsSnCl_3_ materials were highly ductile and possessed lower values of the bulk modulus, which suggests that both the doped samples are suitable to make thin films. The pure CsSnCl_3_ was not capable of achieving the proper utilization of the solar spectrum for photovoltaic conversion due to its large value of band gap. Suitable metal doping in CsSnCl_3_ can enhance the absorptivity as well as the photoconductivity considerably. However, both the Cr- and Mn-doped CsSnCl_3_ showed very much higher absorbance and photoconductivity than the pristine sample due to the creation of an intermediate energy band in the band gap in both the doped CsSnCl_3_ samples. The valence band of Mn-doped CsSnCl_3_ was shifted slightly toward the higher energy region compared with Cr-doped CsSnCl_3_ and this makes the transition of valence electrons to the conduction band from the valence band easier and as a result the Mn-doped sample exhibited higher absorptivity as well as photoconductivity than Cr-doped CsSnCl_3_. The comparative analysis among the different fundamental properties of the pristine and metal-doped CsSnCl_3_ with the metal-doped CsGeCl_3_ and the best entitled inorganic metal halide CsGeI_3_ suggested that CsSn_1−*x*_Mn_*x*_Cl_3_ was the most environmentally friendly perovskite among the available inorganic perovskite materials for application in high-performance solar cells and other potential optoelectronic devices.

## Conflicts of interest

The authors declare no conflicts of interest.

## Supplementary Material
