# Correction: Narrowing band gap and enhanced visible-light absorption of metal-doped non-toxic CsSnCl_3_ metal halides for potential optoelectronic applications

**DOI:** 10.1039/d0ra90054k

**Published:** 2020-05-07

**Authors:** Jakiul Islam, A. K. M. Akther Hossain

**Affiliations:** Department of Physics, Bangladesh University of Engineering and Technology Dhaka-1000 Bangladesh jakiul.pust.phy.39@gmail.com

## Abstract

Correction for ‘Narrowing band gap and enhanced visible-light absorption of metal-doped non-toxic CsSnCl_3_ metal halides for potential optoelectronic applications’ by Jakiul Islam *et al.*, *RSC Adv.*, 2020, **10**, 7817–7827, DOI: 10.1039/C9RA10407K.

The authors regret that there was a mistake in line 8 of the second paragraph of the right hand column of page 7821 of the original article. The text originally read “indirect band gap was 3.15 eV”. The corrected text should read “indirect band gap was 0.315 eV”.

The Royal Society of Chemistry apologises for these errors and any consequent inconvenience to authors and readers.

## Supplementary Material

